# MytiBase: a knowledgebase of mussel (*M. galloprovincialis*) transcribed sequences

**DOI:** 10.1186/1471-2164-10-72

**Published:** 2009-02-09

**Authors:** Paola Venier, Cristiano De Pittà, Filippo Bernante, Laura Varotto, Barbara De Nardi, Giuseppe Bovo, Philippe Roch, Beatriz Novoa, Antonio Figueras, Alberto Pallavicini, Gerolamo Lanfranchi

**Affiliations:** 1 Department of Biology, University of Padova, Via U Bassi, 58/B, 35121, Padova, Italy; 2 C.R.I.B.I. Biotechnology Centre, University of Padova, Via U Bassi, 58/B, 35121, Padova, Italy; 3 Council for Research and Experimentation in Agriculture, Viale XXVIII Aprile, 26, 31015 Conegliano, Treviso, Italy; 4 Institue of Veterinary Sciences (IZSVe), Viale dell'Università, 10, 35020 Legnaro, Padova, Italy; 5 Lagoon Ecosystems UMR 5119, University of Montpellier 2, cc093, place E Bataillon, F-34095 Montpellier cedex 05, France; 6 Institute of Marine Research, CSIC, C/Eduardo Cabello, 6, E-36208 Vigo, Spain; 7 Department of Biology, University of Trieste, P.le Valmaura, 9, 34148 Trieste, Italy

## Abstract

**Background:**

Although Bivalves are among the most studied marine organisms due to their ecological role, economic importance and use in pollution biomonitoring, very little information is available on the genome sequences of mussels. This study reports the functional analysis of a large-scale Expressed Sequence Tag (EST) sequencing from different tissues of *Mytilus galloprovincialis *(the Mediterranean mussel) challenged with toxic pollutants, temperature and potentially pathogenic bacteria.

**Results:**

We have constructed and sequenced seventeen cDNA libraries from different Mediterranean mussel tissues: gills, digestive gland, foot, anterior and posterior adductor muscle, mantle and haemocytes. A total of 24,939 clones were sequenced from these libraries generating 18,788 high-quality ESTs which were assembled into 2,446 overlapping clusters and 4,666 singletons resulting in a total of 7,112 non-redundant sequences. In particular, a high-quality normalized cDNA library (Nor01) was constructed as determined by the high rate of gene discovery (65.6%). Bioinformatic screening of the non-redundant *M. galloprovincialis *sequences identified 159 microsatellite-containing ESTs. Clusters, consensuses, related similarities and gene ontology searches have been organized in a dedicated, searchable database .

**Conclusion:**

We defined the first species-specific catalogue of *M. galloprovincialis *ESTs including 7,112 unique transcribed sequences. Putative microsatellite markers were identified. This annotated catalogue represents a valuable platform for expression studies, marker validation and genetic linkage analysis for investigations in the biology of Mediterranean mussels.

## Background

The marine mussel (*Mytilus galloprovincialis*, Lamark 1819) is commonly found in the Mediterranean Sea, Black Sea, and also intermixed with *M. edulis *along the Atlantic coasts of France, Britain and Ireland [1]. Mussels are suspension feeders commonly living in dense masses at the intertidal and subtidal level, attached among themselves and to hard substrata by the fibrous threads of the byssus. As filter feeders, they are functionally linked with primary producers (mainly phytoplankton and bacteria), and also act as calcium and carbon accumulators, which they use for shell construction. *Mytilus *spp. combine a significant economic importance [2], and an equally relevant role as sentinel species for pollution in coastal waters in many areas of the world [3]. Sessile mussels accumulate various water contaminants in their tissues hence react to environmental changes caused by natural and anthropogenic factors [4] with an assortment of physiological and genetic mechanisms, partly traceable with appropriated tests [5,6].

The DNA content of the haploid genome among bivalves varies from 0.65 to 5.4 pg, it is organized in a number of chromosomes that ranges from 10 to 23; chromosomes tend to be homogeneous in size [7]. *Mytilus galloprovincialis *exhibits a diploid complement of 28 chromosomes and a DNA content estimated in 1.41–1.92 pg [8]. Knowledge on the molecular bases of fundamental bivalve processes, such as the regulation of growth and differentiation or sexual maturation, is still very poor and it is limited by the lack of information about their genes and genomes. Considering all orders in the class of Bivalvia, not a single genome has been fully sequenced yet. The December 2008 release of SRS browser (EMBL Release 97) contains 151,292 nucleotide and 5,334 protein sequences (UniProtKB Release 14.6) (Table 1), with a high rate of redundancy. Most of the sequencing effort was restricted to some bivalve species that appear among the "top 12" aquacultured organisms at a global scale [9]: 45,963 entries came from the Pacific oyster *C. gigas *and the eastern oyster *C. virginica*, 56,091 for the genus *Mytilus *and 10,599 for the venerid *V. decussatus *and *V. philippinarum*. The sequence data available for *Mytilus *spp. are largely insufficient if compared to the number of entries for Pacific white shrimp *L. vannamei *(156,833), for the flat porcelain crab *P. cinctipes *(97,809) and for the *Daphnia sp*. (168,447), which are the most studied crustaceans. As concerns whole genomes, only the commercial oysters *C. virginica *and *C. gigas *have been subjected to BAC library construction [10] whereas the Sea Urchin *Strongylocentrotus purpuratus *[11] and the starlet sea anemone *Nematostella vectensis *[12] have been completely sequenced.

**Table 1:** Nucleotide and protein sequences belonging to all orders of the Bivalvia class.

**Subclass**	**Order**	**Superfamily**	**Family**	**Genus**	**Nucleotide**	**Protein**
Anomalodesmata	Pholadomyoida				**87**	**16**

Heteroconchia	Myoida				**188**	**39**
		Myoidea	Myidae	Mya	40	13
		Hiatelloidea	Hiatellidae	Panopea	24	0

Heteroconchia	Veneroida				**19,585**	**1,038**
		Cardioidea	Cardiidae	Cerastoderma	192	8
		Solenoidea	Pharidae	Ensis	196	9
		Solenoidea	Solenidae	Solen	37	3
		Mactroidea	Mactridae	Lutraria	3	0
		Mactroidea	Mactridae	Spisula	212	31
		Veneroidea	Veneridae	Mercenaria	195	16
		Veneroidea	Veneridae	Venerupis	10,632	124
		Veneroidea	Veneridae	Venus	18	6

Palaeoheterodonta	Trigonioida				**11**	**2**
	
	Unionoida				**7,417**	**975**

Protobranchia	Nuculoida				**164**	**39**
	
	Solemyoida				**35**	**6**

Pteriomorphia	Arcoida				**613**	**168**
		Arcoidea	Arcidae	All *genera*	439	145
		Arcoidea	Glycymerididae	Glycymeris	20	4
	
	Limoida				**146**	**31**
	
	Mytiloida	Mytiloidea	Mytilidae	All *genera*	**57,871**	**1,630**
	
	Ostreoida				**46,668**	**799**
		Ostreoidea	Ostreidae	Ostrea	274	33
		Ostreoidea	Ostreidae	Crassostrea	45,963	726
	
	Pectinoida				**17,456**	**417**
	
	Pterioida				**1,051**	**174**

**Total**					**151,292**	**5,334**

Data available from public databases at December 2008 (EMBL Release 97; UniprotKB Release 14.6). The table is expanded until the genus level only for the most important commercial bivalvia species. The total number of nucleotide and protein sequences for each order is indicated in bold.

Among mussel species, a high number of sequences are accessible for *M. californianus *(43,188) whereas the blue mussel *M. edulis *and the Mediterranean mussel *M. galloprovincialis *have 5,938 and 6,190 nucleotide sequences, respectively. Furthermore, only 409 amino acid sequences with a high rate of redundancy are present for *M. galloprovincialis*. They identify key proteins and enzymes of oxidative phosphorylation (NADH dehydrogenase subunit 1, 2, 3, 4, 4L, 5, 6; ATP synthase F0 subunit 6; cytochrome oxidase subunit I, II, III, cytochrome b), defence mechanisms (defensin MGD-1 precursor; myticin-A, B, C precursor; cathepsin L; lysozyme; MGD2 antimicrobial peptide precursor), adhesion and motility processes (twitchin; adhesive plaque matrix proteins; thread matrix proteins; precollagen-D; actin; paramyosin; tropomyosin; catchin) and stress response (methallothionein 20, 10B, 10 IIIB; heat shock protein 70, 27). Of note, the vitelline coat lysin M7, a protein found in sperm acrosomes of mussels that dissolves the egg vitelline coat permitting fertilization [13], is represented in public databases with a very high rate of redundancy (63 entries).

Bivalve mitochondrial sequences are exceedingly abundant in public databases, if we consider that they represent only a very small fraction of the total DNA and gene content. There are 16 complete (or nearly complete) mitochondrial DNA sequences: 1 Myoida, 3 Veneroida, 2 Unionoida, 3 Mytiloida, 3 Ostreoida and 4 of Pectinoida (Table 2) [14-24]. The mitochondrial component of the genome is particularly interesting in bivalves because some species show a peculiar inheritance called "doubly uniparental" (DUI). Species with DUI have two types of mitochondrial genomes, F and M, which are transmitted through female and male individuals respectively, with males being heteroplasmic and females homoplasmic for F [25-27]. In *M. galloprovincialis*, two mitochondrial DNA lineages (16,744 nt) have diverged by about 20% in nucleotide sequence but preserved identical gene content and arrangement [17].

**Table 2:** List of complete or nearly complete mitochondrial DNA sequences of the class Bivalvia.

**#**	**Subclass**	**Order**	**Organism**	**Refseq**	**Length (nt)**	**Reference**
1	Heteroconchia	Myoida	*Hiatella arctica*	NC_008451	18,244	[14]
2	Heteroconchia	Veneroida	*Sinonovacula constricta*	NC_011075	17,225	Unpublished
3	Heteroconchia	Veneroida	*Acanthocardia tuberculata*	NC_008452	16,104	[14]
4	Heteroconchia	Veneroida	*Venerupis (Ruditapes) philippinarum*	NC_003354	22,676	Unpublished
5	Palaeoheterodonta	Unionoida	*Lampsilis ornata*	NC_005335	16,060	[15]
6	Palaeoheterodonta	Unionoida	*Hyriopsis cumingii*	NC_011763	15,954	Unpublished
7	Pteriomorphia	Mytiloida	*Mytilus galloprovincialis*	NC_006886	16,744	[16,17]
8	Pteriomorphia	Mytiloida	*Mytilus edulis*	NC_006161	16,740	[18,19]
9	Pteriomorphia	Mytiloida	*Mytilus trossulus*	NC_007687	18,652	[20]
10	Pteriomorphia	Ostreoida	*Crassostrea virginica*	NC_007175	17,244	[21]
11	Pteriomorphia	Ostreoida	*Crassostrea gigas*	NC_001276	18,224	Unpublished
12	Pteriomorphia	Ostreoida	*Crassostrea hongkongensis*	NC_011518	16,475	[22]
13	Pteriomorphia	Pectinoida	*Mizuhopecten yessoensis*	NC_009081	20,414	[23]
14	Pteriomorphia	Pectinoida	*Placopecten magellanicus*	NC_007234	32,115	[24]
15	Pteriomorphia	Pectinoida	*Mimachlamys nobilis*	NC_011608	17,963	Unpublished
16	Pteriomorphia	Pectinoida	*Argopecten irradians*	NC_009687	16,221	Unpublished

Data available from public databases (Entrez Taxonomy Browser) at January 2009.

Current genomics technologies, like SAGE [28], differential display [29] and systematic sequencing of expressed sequence tags [30], are very useful approaches to rapidly identify protein coding genes on a large scale in model [31] and non-model organisms [32,33]. Moreover, the frequency of a given sequence in the SAGE or cDNA libraries can be related to the relative abundance of the corresponding mRNA, giving an indication of the level of gene expression [34,35]. EST analysis is also an effective approach for the identification of polymorphic cDNA markers such as microsatellites and single nucleotide polymorphisms [36-38].

Several EST collections have already been reported for commercial bivalves [39] but most of the sequencing effort was restricted to the oyster *C. gigas *[40,41] and *C. virginica *[42,43]. The Oyster Genome Consortium has integrated these EST resources for the construction of a publicly available cDNA microarray. This platform was used to evaluate the degree of cross-species hybridization between *C. gigas *and *C. virginica *[44]. Different genomic approaches have been applied also to *M. galloprovincialis*. A number of cDNA libraries obtained from multiple mussel tissues [45] were sequenced. The resulting collection of independent 3'-end ESTs was assembled in *MytArray *1.0 in order to analyze the tissue transcriptional signatures of Mediterranean mussel exposed to chemical mixtures in laboratory and in the Venice lagoon [46]. Recently other cDNA libraries were constructed from haemolymph of immuno-stimulated mussels to better understand their immune response mechanisms [47]. However, the EST resource of *M. galloprovincialis *remains too small compared to other bivalves such as oyster and *M. californianus*. To date, only 6,190 ESTs have been deposited in GenBank for the Mediterranean mussel. The aim of our study was to increase significantly the number of mussel genes in the public database. For this purpose, we have produced and massively sequenced a high-quality normalized cDNA library in order to generate new thousands of non-redundant ESTs and to analyze the ESTs for microsatellites. We have fully functionally annotated these sequences and we present the first knowledgebase of a mussel transcriptome.

## Construction and content

### Tissues samples and RNA purification

Mediterranean mussels (*M. galloprovincialis*) with a maximum shell length of 6–7 cm and mixed sex were obtained from commercial shellfish stocks from Chioggia, Venice, Trieste (North Adriatic Sea, Italy) and Ria de Vigo (Atlantic ocean, Spain). Bivalves were acclimatized in artificial sea water (Italy) and in tanks having an open-circuit of filtered seawater at 15°C with aeration in Vigo (Spain), and then subjected to different challenges. Selected tissues (gills, digestive gland, foot, anterior and posterior adductor muscles and mantle), essential for vital functions and potentially involved in stress responses, were dissected on ice, rapidly rinsed in sterile saline solution, frozen and stored in a large excess of Trizol reagent (Invitrogen, 15 ml for 0.5–1.5 g. of sample) at -80°C.

Haemolymph (1–2 ml) was withdrawn with a disposable syringe from the posterior adductor muscle of each animal treated with a mixture of heat-inactivated bacteria or a solution of poly I:C (Sigma) mimicking viral infection [47]. Haemocytes were collected by centrifugation, lysed in a few ml of Trizol reagent and stored at -80°C.

Frozen tissues were minced and homogenized for 3–5 min using a Diax 900 (Heidolf, Germany) blender. Total RNA was isolated using the Trizol reagent following the manufacturer's instruction and further purified with LiCl 8 M in order to remove glucidic contaminants. All RNA samples were checked for quality by microcapillary electrophoresis (RNA 6000 Nano LabChip, Agilent Bioanalyzer 2100, Agilent Technologies).

### Construction of cDNA libraries

During five years, 17 independent cDNA libraries were constructed in order to identify genes transcribed in Mediterranean mussel (for more details, see Table 3). Initially we have prepared 3'-end cDNA libraries from multiple mussel tissues, named Ese00, Tst00, Tst01, MxT01, MxT02, MxT03, with a uniform size (300–600 bp) tagging each transcript with a unique probe [45,46]. Recently, we have developed a new method using a combination of the SMART protocol (Clontech), exploiting the template-switching effect at the 5'-end and ensuring almost full-length cDNA, and Gateway technology (Invitrogen), allowing unidirectional cloning without enzymatic digestion [33]. Using this protocol we have constructed 10 further cDNA libraries named DiG01, DiG02, GDG01, Gll01, Hae01, Hae02, Hae03, Hae04, Hae05, MxT04 [46].

**Table 3:** Description of Mediterranean mussel cDNA libraries.

**Name**	**Tissue**	**Description**
**DiG01**	Digestive gland	3 days of treatment with okadaic acid (January 2006 – Trieste, Italy)
**DiG02**	Digestive gland	Treatment with heat-inactivated bacteria (June 2005 – Padova, Italy)
**Ese00**	Mixed tissue	Selected tissues: digestive gland, gills, foot, gonads, haemolymph and mantle (October 2000 – Padova, Italy)
**GDG01**	Digestive gland and gills	Treatment with two mixtures of organic compounds and heavy metals (December 2002 – Padova, Italy)
**Gll01**	Gills	Treatment with heat-inactivated bacteria (June 2005 – Padova, Italy)
**Hae01**	Haemolymph	Off-shore control mussels (June 2005 – Padova, Italy)
**Hae02**	Haemolymph	Treatment with heat-inactivated bacteria (June 2005 – Padova, Italy)
**Hae03**	Haemolymph	Treatment with heat-inactivated bacteria (June 2005 – Vigo, Spain)
**Hae04**	Haemolymph	Treatment with a solution of poly I:C mimicking viral infection (June 2005 – Vigo, Spain)
**Hae05**	Haemolymph	Control mussels (June 2005 – Vigo, Spain)
**MxT01**	Mixed tissue	Gills, digestive gland, foot, anterior and posterior adductor muscles and mantle (June 2002 – Padova, Italy)
**MxT02**	Mixed tissue	Gills, digestive gland, foot, anterior and posterior adductor muscles, mantle and haemolymph (October 2002 – Padova, Italy)
**MxT03**	Mixed tissue	Selected tissues: gills, digestive gland, foot, anterior and posterior adductor muscles, mantle and haemolymph (October 2002 – Padova, Italy)
**MxT04**	Mixed tissue	Treatment with heat-inactivated bacteria (June 2005 – Padova, Italy and Vigo, Spain)
**Nor01**	Mixed tissue	Equal amount (333 ng) of cDNA from DiG01, DiG02, GDG01, Hae01, Hae02, Hae03, Hae04, Gll01, MxT04 have been pooled (April 2006 – Padova, Italy)
**Tst00**	Mixed tissue	Gills, digestive gland, foot, anterior and posterior adductor muscles and mantle (October 2000 – Padova, Italy)
**Tst01**	Mixed tissue	Gills, digestive gland, foot, anterior and posterior adductor muscles and mantle (November 2000 – Padova, Italy)

A normalized *M. galloprovincialis *library (Nor01) was produced to optimize the discovery rate of the random sequencing process by equilibrating the final representation of abundant and rare transcripts. This library was constructed by pooling equal amounts (333 ng) of cDNA from DiG01, DiG02, GDG01, Hae01, Hae02, Hae03, Hae04, Gll01, MxT04 libraries. This cDNA pool was concentrated by Microcon YM 30 (Millipore) and adjusted to a final concentration of 70 ng/μl. For cDNA normalization, 3 μl (about 200 ng) of purified double-strand cDNA plus 1 μl 4× Hybridization Buffer (200 mM HEPES-HCl, pH 8.0; 2 M NaCl) was overlaid with mineral oil, denatured at 98°C for 2 min and then allowed to anneal at 68°C for 5 h. The following pre-heated reagents were then added to the hybridization reaction kept at 68°C: 3.5 μl milliQ water; 1 μl of 5× DNAse buffer (500 mM Tris-HCl, pH 8.0; 50 mM MgCl2, 10 mM DTT); 0.5 μl double-strand nuclease (DSN) enzyme. After further incubation at 68°C for 30 min., the DSN enzyme was inactivated by adding 10 μl of 5 mM EDTA at 68°C for 10 min. The normalized cDNAs samples were diluted with 20 μl milliQ water and used for PCR amplification. The PCR reaction (50 μl) contained 1 μl diluted cDNA, 1 × Advantage 2 reaction buffer (BD Biosciences Clontech), 200 μM dNTPs, 0.15 μM attB1 and attB2 primers, 1 × Advantage 2 Polymerize mix (BD Biosciences Clontech). The amplification protocol consists of 21 cycles of the following consecutive steps: 7 s at 95°C, 20 s at 65°C and 3 min at 72°C. The amplified normalized cDNA was size-selected on SizeSep 400 Spun Columns (GE Healthcare) and directionally cloned into pDONR221 vector (Invitrogen) through BP recombinase.

### DNA sequencing

The systematic sequencing of most recently produced cDNA libraries (DiG01, DiG02, GDG01, Gll01, Hae01, Hae02, Hae03, Hae04, Hae05, MxT04 and Nor01) was performed at the Sequencing Service of Max-Plank Institute for Molecular Genetics (Berlin, Germany). Libraries were arrayed on 384-well plates and single pass DNA sequencing from plasmids was performed by using the vector specific primer attB1_seq (5'-CTTTGTACAAAAAAGCAGGCT-3') and a modified Sanger dideoxy terminator cycle sequencing chemistry, the ABI BigDye kit version 3.1, on Capillary Sequencer systems (Applera ABI 3730 XL and GE Healthcare MegaBase 4500).

### Sequence processing and analysis

Trace2dbest and Partigene [48] were used to process chromatograms, align and clusterize sequences and build an annotation database. Trace2dbest extracts sequences and quality information from traces (Phred algorithm), removes vector contamination and poly(A) and performs the trimming of low quality sequences. Sequences shorter than 150 bp were discarded. Partigene reads all sequence files and performs an assembling process in two step: 1) CLOBB software [49] clusterizes sequences on the basis of BLAST similarity; 2) Phrap [50] makes a consensus from each cluster.

Each consensus, converted into FASTA format, was searched locally in nucleotides database, downloaded from NCBI [51] and UniProtKB sources [52], using Blast-N and Blast-X, respectively. The first 5 High Scoring Pairs from each Blast result were collected and stored in a local PostgreSQL table as a collection of automatic annotations.

Each single annotation in our database was further manually examined to assign the best describing text to the correspondent cluster. Similarities with expectations values greater than e^-6 ^for protein (Blast-X) and e-^40 ^for nucleotide (Blast-N) were considered as poorly informative. Moreover, putative peptides identified by Prot4EST [53] where searched for protein domain in all available protein signature databases by means of InterproScan [54]. Clusters, consensuses and related similarities were electronically organized and stored in a dedicated PostgreSQL database [55].

### Gene Ontology annotation

To each UniProt ID taken from Blast-X description field, we have associated specific Gene Ontology annotations (GO) that integrate information about process, function, and component. The distribution of sequences in each of the main ontology categories was examined and percentages of unique sequences in each of the assigned GO terms was computed. In each of the three main categories of GO, namely Biological process, Molecular function, and Cellular component [56], 100% was considered as the total number of unique sequences having an assigned GO term. Thus, in each main category the percentages do not reach 100% because some deduced proteins result with more than one GO category assigned to them [43].

### Identification of microsatellite containing ESTs

The unique consensus sequences were screened for microsatellites by using the MISA software [57]. Only di-, tri-, tetra-, penta- and esanucleotide repeats were targeted, since mononucleotide repeats are not useful for mapping or population genetics due to difficulties in their genotyping. Strings of oligonucleotide sequences were used to search for microsatellites: 6 repeats for dinucleotide; 5 repeats for trinucleotide; 5 repeats for tetranucleotide, pentanucleotide and esanucleotide.

## Utility and discussion

### General characteristics of the cDNA libraries and EST assembly

Table 3 describes the cDNA libraries prepared from different *M. galloprovincialis *tissues that were used for this project. A total of 24,939 ESTs were subjected to quality examination and 6,151 ESTs were discarded. From the remaining 18,788 (75.33%) high-quality ESTs vector sequences were recognized and deleted. This processed collection of *M. galloprovincialis *ESTs has been deposited in the EBI-GenBank-DBJ database and GenBank accession numbers for each EST are linked in our web site. These ESTs were finally assembled by similarity using bioinformatic tools into 2,446 clusters and 4,666 singletons, resulting in a total of 7,112 non-redundant sequences (consensus). EST production and processing results for each cDNA library is presented in Table 4. The overall average redundancy of ESTs was 73.9%. The lowest level of redundancy was found in the Nor01 library (34.4%) suggesting that the protocol adopted for the construction of this library resulted in a significant normalization of mussel mRNAs. Furthermore, the clustering of ESTs obtained from this specific library resulted in 38.9% of the clusters composed by ≥ 2 ESTs (2,137 hits) in contrast to the 65.6% of singletons (3,359 hits), showing that no definite transcripts are particularly enriched in Nor01 library. Using a subtractive method Li and colleagues have obtained a similar gene discovery rate (about 71%) in catfish ESTs project indicating very low levels of redundancy in both cases [58]. cDNA libraries constructed from a strictly committed tissue showed instead lower percentages of ESTs putatively identifying new transcripts (% discovery), ranging from 9.6% to 21.4%. For example, in the Hae series of cDNA libraries that were produced from mussel haemolymph the transcript discovery rate is uniformly low, with percentage of singletons varying from 18% to 24%. The putative identification of new transcripts was increased of about 27% by the systematic sequencing of Nor01 with respect to MxT03 cDNA library prepared from the main mussel tissues and used to generate the first mussel microarray platform named *MytArray *1.0 [46]. The normalized cDNA library is therefore a very relevant tool for mussel genomics and can be further exploited as an effective source of novel *M. galloprovincialis *mRNAs.

**Table 4:** Results of EST assembly for each Mediterranean mussel cDNA library.

**Library**	**Total ESTs**	**Discared ESTs**	**Analyzed ESTs**	**# EST in cluster**	**# clusters**	**# singletons**	**# consensus**	**% poly(A) detection**	**% gene discovery**	**% redundancy**
**DiG01**	93	24	69	54	31	15	46	66.7	21.7	78.3
**DiG02**	95	23	72	57	28	15	43	73.6	23.6	76.4
**Ese00**	285	121	164	143	90	21	111	34.8	12.8	87.2
**GDG01**	35	10	25	19	19	6	25	64.0	24.0	76.0
**Gll01**	95	18	77	45	37	32	69	51.9	41.6	58.4
**Hae01**	656	93	563	511	237	52	289	56.0	10.3	89.7
**Hae02**	540	100	440	352	196	88	284	41.4	21.4	78.6
**Hae03**	523	130	393	354	126	39	165	83.2	10.9	89.1
**Hae04**	568	116	452	411	130	41	171	81.2	9.5	90.5
**Hae05**	475	134	341	309	110	32	142	83.3	10.0	90.0
**MxT01**	767	200	567	381	260	186	446	34.0	41.1	58.9
**MxT02**	768	577	191	138	90	53	143	39.8	37.2	62.8
**MxT03**	4224	1478	2746	2038	586	708	1294	50.6	38.2	61.8
**MxT04**	74	22	52	52	50	0	50	61.5	0.0	100.0
**Nor01**	15621	3034	12587	9228	2137	3359	5496	23.3	65.6	34.4
**Tst00**	96	55	41	25	24	16	40	53.7	39.0	61.0
**Tst01**	24	16	8	5	5	3	8	25.0	37.5	62.5
**Overall**	24939	6151	18788	14122	4156	4666	8822	54.3	26.1	73.9

Total ESTs = number of produced chromatograms; Discarded ESTs = number of low quality ESTs; Analyzed ESTs = number of sequences processed for clustering; # EST in cluster = number of sequences in cluster; # clusters = total number of clusters; # singletons = number of putative transcripts identified by one single EST; # consensus = number of non-redundant sequences; % poly(A) detection = percentage of sequences where poly(A) has been identified and trimmed; % gene discovery = percentage of ESTs identifying putative new transcripts in the total EST analyzed from each library; % redundancy = percentage of sequences also identified by other cDNA libraries.

**Figure 1:**
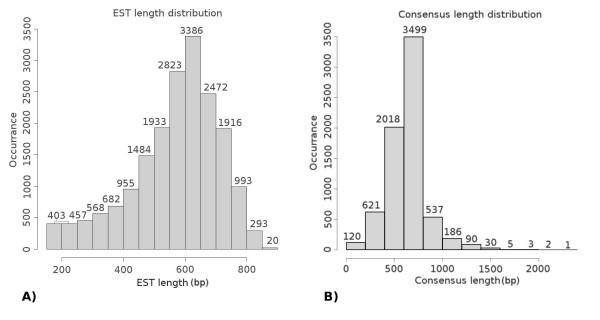
**Representation of length distribution of EST (A) and consensus sequences (B)**.

The number of ESTs in clusters varies from 2 (1,062 clusters) to 190 (1 cluster, MGC10002). Clearly, most of the clusters have a limited number of sequences, that represent the high efficiency of normalization. The average length of cluster is 638 bp with the longest assembled sequence being 2,290 bp (MGC10001: *Mytilus galloprovincialis mitochondrion, complete genome*) and the shortest 153 bp (Figure 1). The detection of a polyadenylated tail at the 3' end of clusters for each cDNA library varied from 23% of the normalized cDNA library (Nor01) to 50% of MxT03. The low percentage of cluster consensuses containing the 3'-end region in Nor01 is due to the great enrichment in full-length cDNAs obtained with the SMART technology [33] used for library construction.

We have decided to sequence intensely only the two cDNA libraries (MxT03, Nor01) that could contain a more general information of mussel transcriptome, since they were constructed by pooling equal amounts of RNA from different tissues (gills, digestive gland, foot, anterior and posterior adductor muscles and mantle). Instead, others cDNA libraries constructed from single tissues (DiG01, DiG02, Hae00, Hae05) or from mussels treated with organic compounds and heavy metals (GDG01) or heat-inactivated bacteria (Gll01, Hae01, Hae02, Hae03, Hae04) have been sequenced to a less extent because of the more committed nature of their transcriptome.

In the next future we are planning to apply a round of large-scale sequencing using second-generation of high throughput DNA sequencers (e.g. Roche 454) to the normalized cDNA library to fully exploit the information contained in it.

### Functional annotation of ESTs and construction of the *M. galloprovincialis *transcript catalogue

**Figure 2:**
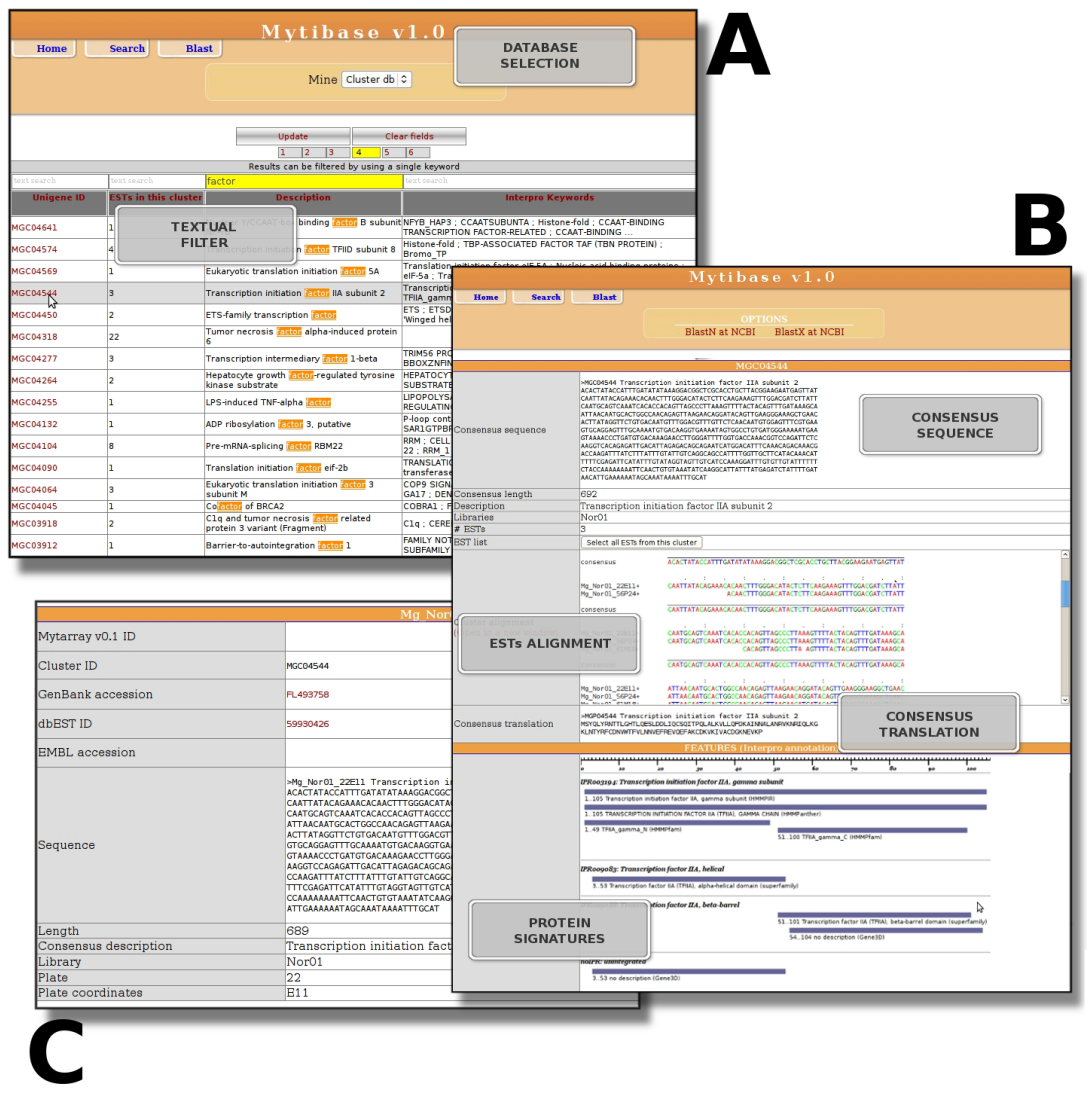
**Snapshots of the MytiBase web interface**. The search interface (A) allows users to filter data fields, (i.e. number of ESTS in a cluster, or words in cluster description). Following the cluster/EST link, a detailed report (B and C) can be accessed.

In order to make an assessment for the putative identities of the ESTs, each non redundant consensus sequence was searched in the public nucleotide and UniProtKB databases using Blast-N and Blast-X with an *e*-value cut off of < e^-40 ^and < e^-6^, respectively. These values were empirically chosen by taking into account the low amount of genomic data available for *M. galloprovincialis *and similar mussel species, and the need of stringency in providing a reliable catalogue of Mediterranean mussel genes. Additionally, the results of these searches were manually examined in order to assign the best describing annotation to EST clusters. The sequencing and annotation data have been organized in a user-friendly, integrated database called MytiBase that is available from our web site [55]. It provides several tools to search cleaned and assembled EST sequences, genes and GO annotations. The user may input and submit keywords or IDs to the server using the web interface and results are sent back in proper formats such as shown in Figure 2. MytiBase also provides a complete view of cluster consensus sequences, BLAST searches and InterPro domains.

Overall, 54% of non-redundant sequences (3,837 out of 7,112) identified by about 40% of total ESTs (7,694 out of 18,788) showed no or poor similarity with publicly available sequences. These unknown mussel transcripts support the discovery of new genes, and possibly new gene networks and metabolic pathways in mussel. Interestingly, three unknown transcripts are among the 20 most expressed genes: MGC00007 (188 ESTs), MGC00293 (61 ESTs) and MGC00279 (54 ESTs). However, we realize that some ESTs are relatively short and falling within the 3'-untranslated regions, thus their identities could probably not be easily revealed by sequence similarity comparison [7]. A large number of ESTs with no similarity hit is a common feature of studies on mollusk species [39,59,60], probably because of the great level of amino acid divergence found between invertebrates and the reference taxa currently used in genomics.

**Figure 3:**
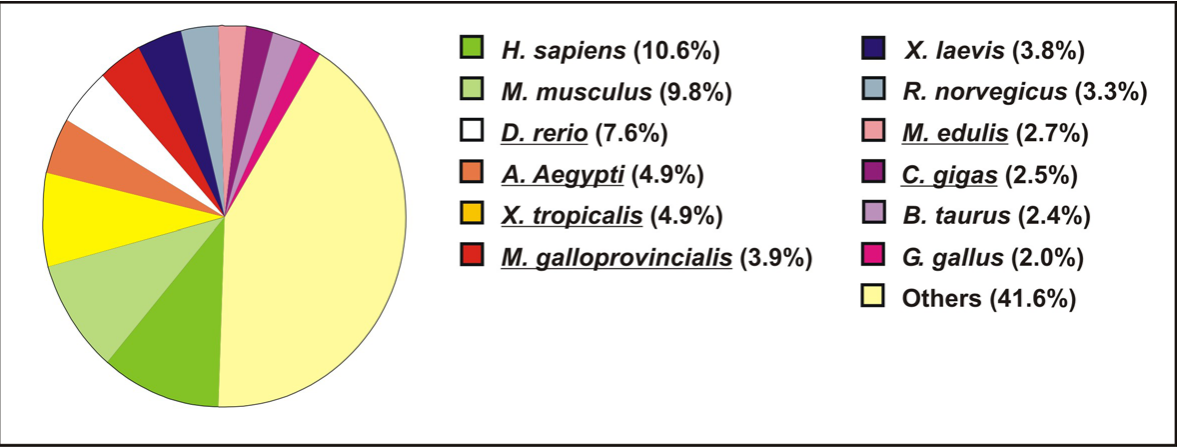
**Organisms most represented in the protein similarity searches with mussel sequences**. Percentages of transcripts finding Blast-X similarity (E-value < e^-6^) in the non-redundant protein database attributable to a given organism (manually examined annotations). The organism names underlined are those with statistically significant (*p*-value = 0) representation in our MytiBase respect to the number of protein sequences in UniProtKB database.

Figure 3 summarizes the non-redundant sequences that show similarity to known *M. galloprovincialis *genes and those showing a significant rate of similarity to predicted proteins from various organisms. The complete list of these sequences (3,275, 46% of total consensuses) is reported in the Additional File 1. Only 7.3% were most similar to known sequences from the genus *Mytilus (M. californianus, M. edulis, M. galloprovincialis, M. trossulus*), which could be due to the limited number of *Mytilus *gene and protein sequences in the public databases (56,079 nucleotide and 1,185 protein sequences at December 2008). The Additional file 1 also shows that mussel transcripts referred to diverse mitochondrial genes (*ATP synthase a*, *Cyt b*, *cyt c*, *COI*, *COII*, *COIII*, *ND1*, *ND2*, *ND3*, *ND4*, *ND5*, *ND6*) are represented in our EST catalogue (416 out of 18,788) and they are annotated as "*Mytilus galloprovincialis *mitochondrion, complete genome".

A significant number of stress-, immune-, and defense-related transcripts were putatively identified from the systematic sequencing of five haemocyte cDNA libraries from healthy (Hae01, Hae05) bacterial-treated (Hae02, Hae03) or poly I:C treated mussels (Hae04). Small cationic antimicrobial peptides (AMPs) such as *Myticin *A, *Myticin *C, *Mytilin *B and *Mytilin *C were highly represented in our EST catalogue (0.4%, 0.8%, 1.0%, and 0.7% of total ESTs, respectively). We have also identified different types of lectins (C-type lectin, sialic acid binding lectin, fucolectin, galectin) that are molecules mediating agglutination processes acting as opsonins [61], thus playing a decisive role in the humoral defense against pathogenic organisms. Studies on the expression of these genes could improve the general understanding of the innate immune response and defense mechanisms in mussels.

Mussel of the *Mytilus *genus are sessile shells and, interestingly, all collagen precursors of the adhesive apparatus (byssus) such as the *proximal collagen *(PreCol-P), *distal collagen *(PreCol-D) and *pepsin-resistant nongradient collagen *(PreCol-NG) have been identified in our libraries. The elastic domains of PreCol-P, the silk fibroin-like domains of PreCol-D, and the plant cell wall-like domains of PreCol-NG characterize the unique, collagenous block copolymer found in the byssus threads of mussel. Moreover, we identified three members of the protein family secreted by the mussel foot (*Mgfp3, Mgfp4, Mgfp6*), that are located in the adhesive plaque, providing adhesiveness and strength to the fibrous collagen core of byssus threads [62].

The 3,837 consensus sequences with no significant similarities were locally searched for conserved protein domains using the InterPro tool [54] in order to find some clues for their possible biological role and generally to identify interesting candidates for functional studies in the next future. Interestingly, this approach has led us to the identification of 26 transcripts containing universal stress protein-like domains (Usp) and two transcripts (MGC03893 and MGC01634) that present a domain conserved in a number of proteins involved in heavy metal transport or detoxification [63]. The study of these transcripts could be useful to increase the knowledge about the physiological and genetic mechanisms activated by mussels in response to toxic pollutants (heavy metals in particular).

### Annotation of *M. galloprovincialis *ESTs

**Figure 4:**
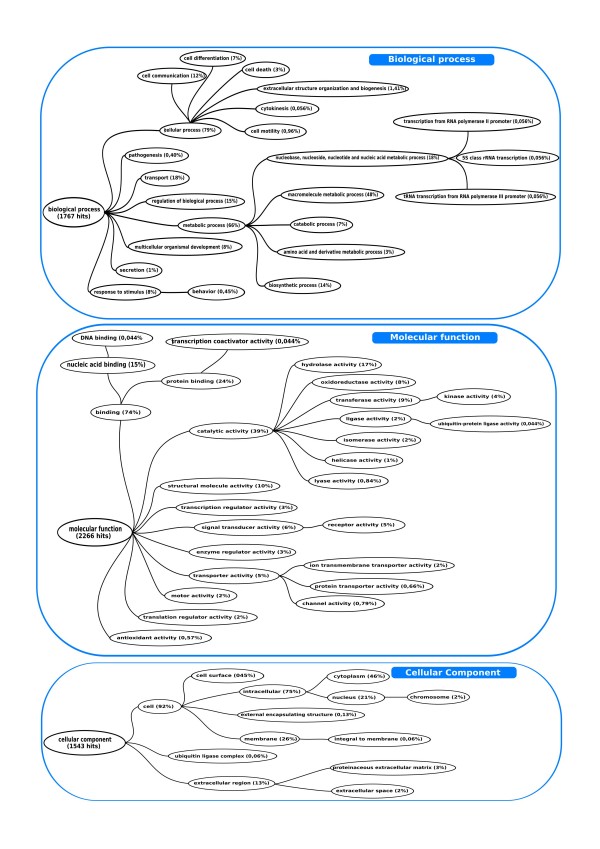
**Gene Ontology categorization of 3,275 *M. galloprovincialis *annotated sequences**. The total numbers of consensus sequences classified in each main GO category are 1,767 for *Biological Process*, 2,266 for *Molecular Function *and 1,543 for *Cellular component*. Since a gene product could be assigned to more than one GO term, the percentages in each main category do not add up to 100%. See the Additional files 3, 4 and 5 for more details.

Gene Ontology (GO) has been widely used to perform gene classification and functional annotation [64] using controlled vocabulary and hierarchy including molecular function, biological process and cellular components. GO categories were assigned to 3,275 *M. galloprovincialis *sequences with a significant Blast-X hit, using generic GO slim which are cut-down versions of the GO ontologies containing a subset of the terms of the whole Gene Ontology. These slim annotations give a broad overview of the ontology content without the details of the specific fine grained terms. Figure 4 shows the distribution of gene ontology terms according to generic GO slims (Additional files 3, 4 and 5). "Cellular process" (79%) resulted the most dominant term out of the 1,767 consensus sequences which were annotated to the Biological Process in GO slims. In this subcategory we found genes involved in cell communication (12%), cell differentiation (7%), cell death (3%) and cell motility (1%). We putatively identified five members of the cysteine-aspartic acid protease (caspase) family (*caspase-1, -2, -7, -8, -9*), that plays a central role in the execution-phase of cell apoptosis [65] and a family of proteins that inhibits apoptosis by binding to tumor necrosis factor receptor-associated factors like *inhibitor of apoptosis 1 *and *3*. We also found genes belonging to the Bcl-2 protein family that acts as anti- or pro-apoptotic regulator involved in a wide variety of cellular activities. *Bcl-2 *encodes an integral outer mitochondrial membrane protein that blocks the apoptotic death whereas *BCL2-associated X protein *(Bax) is pro-apoptotic and accelerates S-phase progression [66]. In the "Cell communication" subcategory we have identified three members of the *Notch homolog, translocation-associated *(*Notch*) family (*Notch 1, 2 and 3*). These genes play a key role in a variety of developmental processes by controlling cell fate. The Notch signaling network is a fundamental and evolutionarily conserved intercellular signaling pathway which regulates interactions between physically adjacent cells [67]. Interestingly, various members of the Rab family (*Rab5, Rab5A, Rab6, RabGAP/TBC, Rab27A, Rab32*) have been putatively identified, confirming the hypothesis that a number of Rab GTPases are conserved from yeast to humans. The different Rab GTPases are localized to the cytosolic face of specific intracellular membranes, where they function as regulators of distinct steps in membrane traffic pathways such as vesicle formation, actin- and tubulin-dependent vesicle movement, and membrane fusion [68].

"Metabolic process" was the second most abundant GO subcategory in *Biological Process*, with 66% of annotated mussel transcripts assigned to it. A large majority of transcripts (48%) belonging to macromolecule metabolic process showed putative identity with ribosomal sequences and genes connected to the translation machinery. We found genes with regulative functions in the translational initiation, like *translation factor SUI1*, *initiation factor 2A, 3D, 3E, 3M, 4E, 5A *and elongation, like *elongation factor 1α, 1γ, 2, Ts *(mitochondrial). We were also able to identify some ubiquitin protein ligases (*Bre1, MIB1, NRDP1, RING2, RNF19A, SIAH1, UBR2, UBR3, UBR5*) and two members of the proteasome complex (*subunit α and β*) involved respectively in tagging and degradation of unneeded or damaged proteins [69]. Other mussel transcripts appear to be involved in the mechanisms of DNA transcription (*DNA polymerase II, mediator of RNA polymerase II, transcription elongation factor B, SPT5, SPT6, transcription initiation factor IIA, IID (subunit 1, 8, 9, 11) and transcription intermediary factor 1 β*).

Eight percent of the annotated transcripts were assigned to the Biological Process subcategory named "response to stimulus". This class includes a set of genes recruited in stress responses and potentially useful in environmental studies such as *heat shock proteins *(*HSP25, HSP60, HSP70, HSP71, HSP90*), *metallothioneins (MT10III, MT20II)*, *ferritin*, *cytochrome *P450 and *glutathione S-transferase *(*GST*). Ferritin in fact plays a key role in the metabolism of cellular iron including storage and detoxification [70,71] and it is also involved in shell formation by iron storage [72]. Metallothioneins are ubiquitous metal-binding proteins that function in the homeostasis of essential metals, such as zinc and copper, as well in detoxification mechanisms by sequestering toxic metals such as cadmium, lead, and mercury [73,74]. Cytochrome P450 isoforms are involved in the metabolism of xenobiotics, such as polycyclic aromatic hydrocarbons [75,76] while GST isoforms are important in the metabolism of organochlorinated compounds and other chemicals [77]. Both enzymes have been used in mollusks as biomarkers for the assessment of coastal water contaminated by these pollutants [78]. The systematic sequencing also identified some antioxidant enzymes such as *thioredoxin, thioredoxin reductase*, *glutathione peroxidase *and *superoxide dismutase *that are involved in the oxidative stress responses [79].

Some annotated transcripts such as *matrix metalloproteinase 1*, *metalloproteinase inhibitor 2*, and *metalloproteinase inhibitor 3*, were classified in the *organism development *GO subcategory. They are involved in the breakdown of extracellular matrix in normal physiological processes, such as embryonic development, reproduction, and tissue remodeling, as well as in disease processes and their inhibitors [80].

"Protein binding" resulted as the most common GO term (24%) associated to the 2,266 consensus sequences which were assigned to the Molecular Function category in GO slims, followed by the "hydrolase" (17%) and "nucleotide binding" (15%) terms.

### Identification of *M. galloprovincialis *microsatellites

Simple sequence tandem repeats (SSR), also known as microsatellites, are an excellent source of genetic markers to use in linkage mapping, parentage assignment and population genetics [81]. Among the 7,112 non-redundant sequences examined in this study, we identified 154 (2.2%) consensuses containing SSR by using MISA software [57]. Five of these present 2 or 3 distinct simple sequence repeats interrupted by more than 100 bp for a total of 159 identified SSR. The most frequent motifs are di- (27.7%) and tri- (61.0%) nucleotides with a prevalence of TA (15 out of 44), AT (15 out of 44), AGC (17 out of 97) and CAA (10 out of 97) respectively. The 5' and 3' SSR flanking regions have an average length of about 330 bp and only 33 repeats show flanking region shorter than 50 bp, making the design of primers difficult. A list of all microsatellite-containing ESTs is presented in the Additional file 2. Overall, 62,3% (99 out of 159) of non-redundant sequences containing microsatellites showed no or poor similarity in the nucleotide and protein databases. Sixteen sequences are similar to precollagen protein and share repetitive motifs generally found in this class of proteins [82]. Comparing our microsatellite sequences with those described in a recent work on mussel EST-SSRs [83] we conclude that we have identified about 50 useful markers for genetic studies of mussel populations. Since our novel microsatellite markers were developed on the basis of expressed sequences and they are presumably conserved across other *Mytilus *species, they could also be useful for comparative mapping and for a molecular approach to mussel ecology.

### Comparative analysis of *M. galloprovincialis *and *M. californianus *EST sequences

Recently, Gracey and colleagues have deposited 22,836 5'- and 3'-end ESTs of *Mytilus californianus *in the EBI-GenBank-DBJ database. Taking advantage of this data, we compared the transcribed genomes of *M. californianus *and *M. galloprovincialis *in order to verify the level of divergence between these two occasionally sympatric species (i.e. California Bay, North America). For this purpose, following the process described in the Methods, we have aligned 22,836 *M. californianus *ESTs sequences together with our 18,788 Mediterranean mussel ESTs. Since this process generated only 1,054 hybrid clusters, we can assume that, at nucleotide level, the two mussel datasets seem to be very different. However, the number of hybrid clusters may be influenced by some technical issues such as the different methodology for cDNA library construction and different parameters applied for base calling and trimming of *M. galloprovincialis *or *M. californianus *chromatogram traces. Recently, the full protein sequences for preCol D, NG and P from *M. californianus*, *M. edulis *and *M. galloprovincialis *have been compared. In agreement with our evidences, the preCols from *M. californianus *are more divergent from the other two closely related species [84]. The genetic divergence between *M. californianus *and *M. galloprovincialis *is supported also by the clearly different external morphology and by the lack of cross-hybridization in spite of sympatric localizations, in contrast of *M. trossulus *and *M. galloprovincialis *species [85,86]. Furthermore, analysis of mitochondrial DNA sequences has revealed that *M. californianus *is the most divergent of mussel species, whereas *M. edulis *and *M. galloprovincialis *are the most similar [87]. Analysis of 18S rDNA sequences again showed that *M. californianus *is the most divergent species [88].

## Conclusion

The genome sequence of Mediterranean mussels is not yet available and therefore the systematic sequencing of cDNA libraries of these invertebrates represents a powerful approach to identify large numbers of transcripts that could be used in gene expression and functional genomics studies [89] and also a first step toward the deciphering of the complete mussel genome. We have produced and sequenced 17 cDNA libraries from different *M. galloprovincialis *tissues, obtaining 18,788 high-quality ESTs that identify 7,112 unique transcribed sequences. In particular, a highly effective normalized cDNA library (Nor01) was constructed, as demonstrated by its high gene discovery rate (65.6%). Over 54% of the *M. galloprovincialis *transcribed sequences resulted in no BLAST matches with published sequences and they probably represent novel genes that could be targeted for functional studies. Of the 7,112 unique sequences, the majority (5,400) were novel ESTs for this species. Moreover, the alignment of sequences from *M. californianus *and *M. galloprovincialis *EST collections resulted in only 1,054 clusters composed by ESTs from both species. Despite possible difference in sample origin and sequence processing, this data has two implications. First, despite the evolutionary and geographical vicinity, these two species appear transcriptionally different. Second, global transcriptome analysis in mussels could make use of specie-specific microarray platforms. In oyster species the level of cross-hybridization between *C. gigas *and *C. virginica *was shown to be 30–40% using a microarray platform with sequences derived from cDNA libraries of both species [44]. Therefore, our collection represents a significant addition to the existing genomic resources for the Mediterranean mussel and generally for *Mytilus *species. All sequencing data have been organized in a dedicated database available from our web site [55]. This EST collection is also a potential source for the development of genetic markers including microsatellite and single nucleotide polymorphisms. Among the 7,112 unique sequences, 159 (2%) unique microsatellite containing ESTs were identified by using MISA software. On the basis of the cluster consensus sequences, we are now producing a *M. galloprovincialis *microarray platform with transcript-specific oligonucleotide probes. The information contained in our database will therefore provide a valuable resource for future studies of mussel transcriptional responses to various biological conditions such as environmental challenges [90], morphological development and bacterial or viral infections.

## Availability and requirements

Project name: Generation and analysis of ESTs from the Mediterranean mussel (*Mytilus galloprovincialis*);

Project home page: ;

Operating system(s): Debian GNU/Linux;

Programming language: PHP;

License: none;

Any restrictions to use by non-academics: none. Users can obtain a personal account and full access to MytiBase by free subscription.

## Authors' contributions

PV performed most mussel treatments and work management, participated in total RNA sample preparation, systematic sequencing of cDNA libraries and design of study. CDP participated to systematic sequencing of the cDNA libraries, annotation of ESTs and drafted the manuscript. FB performed bioinformatics analysis of cDNA libraries sequence data, clustering, annotation of ESTs and identification of microsatellite containing ESTs. LV performed total RNA sample preparation and systematic sequencing of the cDNA libraries. BDN participated in development of cDNA libraries production method and constructed the normalized library. GB contributed to the treatment of Italian mussels with bacterial cocktails. PR donated a mixture of dead bacteria (*Micrococcus lysodeikticus, Vibrio splendidus and Vibrio anguillarum*) for the challenge. AF provided haemolymph from mussels treated with heat-inactivated bacteria and a solution of poly I:C mimicking viral infection. AP and GL supervised the study, participating in the design and coordination of the work, the interpretation of data and manuscript writing. All Authors read and approved the final version of the manuscript declaring that they have no potential conflicts of interests.

## Supplementary Material

Additional file 1 **List of *M. galloprovincialis *annotated sequences.** This table lists 3,275 non-redundant sequences identifying known *M. galloprovincialis *genes or sequences showing significant similarity with predicted protein from mollusks and other organisms. The table includes cluster ID, number of ESTs in cluster, Blast annotation, E-value and organism name. Click here for file

Additional file 2 **Microsatellites in *M. galloprovincialis *EST sequences.** This table lists the 159 microsatellites identified in *M. galloprovincialis *EST sequences with MISA software. Click here for file

Additional file 3 **Details of Biological process annotation.** This table provides the detailed information of each annotated sequence (1,767 hits) grouped in Biological process slim. The table includes cluster ID, number of ESTs in cluster, Blast annotation and organism name. Click here for file

Additional file 4 **Details of Molecular function annotation.** This table provides the detailed information of each annotated sequence (2,266 hits) grouped in Molecular function slim. cluster ID, number of ESTs in cluster, Blast annotation and organism name. Click here for file

Additional file 5 **Details of Cellular component annotation.** This table provides the detailed information of each annotated sequence (1,543 hits) grouped in Cellular component slim. cluster ID, number of ESTs in cluster, Blast annotation and organism name. Click here for file
